# Klotho and fibroblast growth factors 19 and 21 serum concentrations in children and adolescents with normal body weight and obesity and their associations with metabolic parameters

**DOI:** 10.1186/s12887-020-02199-2

**Published:** 2020-06-16

**Authors:** Anna Socha-Banasiak, Arkadiusz Michalak, Krzysztof Pacześ, Zuzanna Gaj, Wojciech Fendler, Anna Socha, Ewa Głowacka, Karolina Kapka, Violetta Gołąbek, Elżbieta Czkwianianc

**Affiliations:** 1 Department of Gastroenterology, Allergology and Pediatrics, Polish Mother’s Memorial Hospital-Research Institute, 281/289 Rzgowska St, 93-338 Lodz, Poland; 2 Department of Biostatistics and Translational Medicine, Medical University of Lodz, Mazowiecka 15, 92-215 Lodz, Poland; 3 Center of Medical Laboratory Diagnostics and Screening, Polish Mother’s Memorial Hospital-Research Institute, Rzgowska 281/289, 93-338 Lodz, Poland

**Keywords:** Children, Obesity, Insulin resistance, Metabolic syndrome, Klotho, FGF19, FGF21

## Abstract

**Background:**

Fibroblast growth factor 19 (FGF19), fibroblast growth factor 21 (FGF21) and Klotho are regulators of energy homeostasis. However, in the pediatric population, the relationships between obesity, metabolic disorders and the aforementioned factors have not been clearly investigated. We analyzed the role of FGF19, FGF21 and Klotho protein in children with normal body weight as well as in overweight and obese subjects and explored their associations with insulin resistance (IR) and metabolic syndrome (MS) and its components.

**Methods:**

This was a cross-sectional study conducted in a group of hospitalized children and adolescents. Laboratory investigations included serum analysis of FGF19, FGF21, and Klotho with ELISA kits as well as the analysis of the lipid profile and ALT serum concentrations. Moreover, each subject underwent an oral glucose tolerance test (OGTT) with fasting insulinemia measurement to detect glucose tolerance abnormalities and calculate the Homeostatic Model Assessment of Insulin Resistance (HOMA-IR) index. Furthermore, the clinical analysis included blood pressure measurement, body fat percentage estimation and assessment of the prevalence of MS and its components.

**Results:**

The study was conducted with 174 children/adolescents aged 6–17 years with normal body weight (*N* = 48), obesity (*N* = 92) and overweight (*N* = 34). Klotho concentration was significantly higher in the obese children [median 168.6 pg/ml (90.2 to 375.9)]) than in the overweight [131.3 pg/ml (78.0 to 313.0)] and normal-body-weight subjects [116.6 pg/ml (38.5 to 163.9)] (*p* = 0.0334) and was also significantly higher in insulin-resistant children than in insulin-sensitive children [185.3 pg/ml (102.1 to 398.2) vs 132.6 pg/ml (63.9 to 275.6), *p* = 0.0283]. FGF21 was elevated in patients with MS compared to the FGF21 levels in other subjects [136.2 pg/ml (86.5 to 239.9) vs 82.6 pg/ml (41.8 to 152.4), *p* = 0.0286]. The multivariable model showed that FGF19 was an independent predictor of IR after adjusting for pubertal stage and BMI Z-score.

**Conclusions:**

Klotho levels were associated with body weight status in children and adolescents. Moreover, Klotho, FGF19 and FGF21 concentrations correlated with IR status and/or components of MS.

## Background

Overweight and obesity in children and adolescents have become a worldwide problem [1, 2]. Excessive body mass promotes insulin resistance (IR) in tissues, which increases the risk of type 2 diabetes, metabolic syndrome (MS) and nonalcoholic fatty liver disease (NAFLD). All these conditions contribute to future cardiovascular risk [3] and must be actively addressed. One of the central agents involved in obesity is adipose tissue and its main hormone, adiponectin, which increases insulin sensitivity [4]. However, other signaling molecules, including those not derived from adipocytes, have recently drawn attention in regard to their role in lipid and glucose metabolism [5].

Particular interest has been given to the fibroblast growth factor subfamily 19, which includes fibroblast growth factor 19 (FGF19) and fibroblast growth factor 21 (FGF21). These hormones have been reported to regulate energy homeostasis in the prolonged response to nutritional status after insulin and glucagon action. FGF19 is mainly secreted from the small intestine in response to food intake and exerts insulin-like effects: it promotes glycogen synthesis and inhibits gluconeogenesis. FGF21, on the other hand, is released from the liver in response to starvation and exhibits glucagon-like properties: it promotes lipolysis, thermogenesis and gluconeogenesis [5–7].

It was previously shown that adult patients with obesity and metabolic diseases present reduced serum FGF19 levels with compensatory increases in FGF21 concentrations [7–10]. However, in children and adolescents, the relationships between obesity, metabolic disorders and the aforementioned factors have not been clearly described [11, 12].

The biological action of molecules from the FGF19 subfamily is mediated by a transmembrane Klotho protein, which promotes their binding to specific receptors [13]. Furthermore, the soluble form of the Klotho protein can, itself, act as a hormone that is detectable in blood, urine and cerebrospinal fluid [14, 15]. Klotho is one of the positive regulators of adipogenesis; however, the relationship between nutritional status and the serum concentration of Klotho is not certain [16–19].

The goal of this study was to investigate FGF19, FGF21 and Klotho serum concentrations in children and adolescents in relation to body weight status. We also aimed to evaluate the association between the factors mentioned above and the occurrence of MS and its components. Finally, we assessed the relationship between the concentrations of the measured proteins and IR.

## Methods

### Participants

This was a cross-sectional observational study based on patients aged 6–17 years who were hospitalized between 2015 and 2019 in the Department of Gastroenterology, Allergology and Pediatrics, Polish Mother’s Memorial Hospital – Research Institute in Lodz, Poland due to gastrointestinal tract symptoms. Patients with confirmed organic causes of symptoms were excluded from the study. All obese and overweight subjects (identified by ICD code) were invited to participate in the study. Nonobese children and adolescents were included as a convenience sample (with a guardian’s consent and lack of contraindications to participate). Exclusion criteria included admission due to acute conditions (trauma, infection, exacerbation of chronic disease), chronic inflammatory diseases, chronic kidney diseases, endocrine disorders (e.g., hyper- or hypothyroidism, pituitary hormone deficiency, type 1 diabetes, adrenal insufficiency, Cushing’s syndrome), malignancy and/or current use of antibiotics or other medications that might influence body composition or glucose and lipid metabolism (e.g., thyroid medication, metformin, steroids). Only children born at term and with adequate birth mass were included in the study. During hospitalization, the participants received a standard diet containing 1500–2000 kcal/day (15% protein, 30% lipids, 55% carbohydrates). The energy supply varied according to differences in patient age, sex and body weight [20]. Parents and children ≥16 years old provided written informed consent before participation. The study was approved by a local bioethics committee (PMMH-RI 39/2015).

### Anthropometric measurements, blood pressure and pubertal development assessment

Upon admission to the hospital, all participants underwent measurements of body weight [kg], height [cm] (Radwag WPT 60/150 OW) and waist circumference [cm], as well as subscapular and triceps skinfold thickness [mm] (MSD Skin Fold Meter). Body mass index (BMI) was calculated according to the formula weight/height^2^ and was converted into Z-scores and percentiles based on national growth charts [21, 22]. We used the 85th and 95th BMI percentile cut-offs to divide the study group into participants with normal body weight, overweight and obesity. Body fat % (BF%) was estimated by Slaughter’s equation [23]. However, due to the lack of modern, population-specific growth charts, BF% was not converted into Z-scores. We also decided against standardizing BF% to body surface or other metrics to keep this parameter simple and easily interpretable. During the physical examination, blood pressure (systolic, diastolic) measurements were performed with a standard procedure (auscultatory, aneroid nonmercury manometer) and interpreted using country-specific centile charts [24]. The diagnosis of arterial hypertension was based on three measurements performed on different occasions. Finally, we assessed pubertal development stage using the Tanner scale (from 1 to 5) [25].

### Blood sampling and laboratory analyses

Venous blood samples were collected after 12 h of fasting into standard vacuum tubes on the second day of hospitalization. Low-density lipoprotein cholesterol (LDL-C) was measured directly by a two-step reaction. Triglycerides (TGs), total cholesterol (TC) and high-density lipoprotein cholesterol (HDL-C) were analyzed using enzymatic colorimetric assays. The enzymatic activity of alanine aminotransferase (ALT) was measured by the akinetic method, and plasma glucose was measured by the oxidase method. All these assays were performed using the Vitros 5.1FS or 4600 platforms (Ortho Clinical Diagnostics, USA). Electrochemiluminescence was used to measure serum insulin levels (Cobas e 601, Roche Diagnostics, USA).

Furthermore, each child underwent a standard 2-h oral glucose tolerance test (OGTT) with 1.75 g glucose/kg (max. 75 g). Two hours after ingestion, plasma glucose between 7.8 mmol/L (140 mg/dl) and 11.1 mmol/L (200 mg/dl) were interpreted as impaired glucose tolerance.

Serum samples for FGF19, FGF21 and Klotho analysis were immediately stored at − 80 °C until analysis. They were thawed at room temperature only once for the measurement. We measured FGF19 and FGF21 concentrations with Human FGF19 and FGF21 ELISA Kits (BioVendor, Brno, Czech Republic) according to the manufacturer’s instructions with an ELISA reader iMARK™ (Bio-Rad) at a wavelength of 450 nm. The manufacturer reported no observed cross-reactivity with human FGF19, FGF21 and FGF23. The limits of detection for FGF19 and FGF21 were 4.8 pg/ml and 7.0 pg/ml, respectively.

We used the double-antibody sandwich ELISA Kit to determine serum Klotho concentrations (ELISA Kit for Klotho SEH757Hu, Cloud-Clone Corp, Houston, TX, USA). The analysis was performed as instructed by the manufacturer, with the ELISA reader iMARK™ (Bio-Rad) at a wavelength of 450 nm. The manufacturer reported no significant cross-reactivity or interference between Klotho and analogs. The detection range was 15.6–1000 pg/ml.

We described the levels of FGF19, FGF21 and Klotho below the detection ranges as 0.

### Insulin resistance and metabolic syndrome diagnosis

IR was evaluated by calculating the Homeostatic Model Assessment of Insulin Resistance (HOMA-IR) index according to the following formula: fasting insulinemia (μU/ml) × fasting glycemia (mmol/l)/22.5. Excessive IR was diagnosed when HOMA-IR exceeded 2.67 in boys and 2.22 in girls in the prepubertal period and 5.22 in boys and 3.82 in girls in the pubertal period [26]. MS diagnosis was based on the International Diabetes Federation criteria from 2007: visceral fat obesity (waist circumference ≥ 90th percentile) plus any two of the other four factors: elevated TGs concentration (≥ 150 mg/dl), reduced HDL-C concentration (HDL-C < 40 mg/dl), elevated arterial blood pressure (≥ 95th percentile, systolic ≥130 mmHg, or diastolic ≥85 mmHg), and elevated fasting glycemia (≥ 100 mg/dl) [27]. According to the abovementioned criteria, there were no diagnoses of MS in the group of children younger than 10 years old.

### Statistical analysis

We compared the normal weight, overweight and obese groups in terms of clinical characteristics and concentrations of FGF19, FGF21 and Klotho proteins with Kruskal-Wallis ANOVA with post hoc Dunn tests. The data are presented as medians and 25–75% ranges. The relationships between continuous variables and concentrations of FGF19, FGF21 and Klotho proteins were assessed by Spearman’s R coefficients. Given low variability of continuous variables in our cohort, we decided to interpret Spearman’s correlation coefficients < 0.3 as weak associations. We noted the frequencies of important metabolic outcomes (presence of arterial hypertension, dyslipidemia, MS, etc.) in each group and compared them (with the normal weight group used as reference) using odds ratios with 95% confidence intervals (95% CI). We then compared the concentrations of the investigated proteins in patients with and without specific conditions with Mann-Whitney U tests.

The relationship between the measured protein concentrations and IR was evaluated using multivariate linear regression with HOMA-IR (log-transformed with base 10) as a continuous outcome. The initial predictors included sex, age, Tanner stage, BMI Z-score, BF% and FGF19, FGF21 and Klotho serum concentrations. After the univariate assessment, we discarded BF% due to its high correlation with BMI Z-score. FGF21 and Klotho were also eliminated due to nonsignificant associations with HOMA-IR. Age and sex were retained in the model despite no significant association with the outcome. For the sake of clarity, physical development (Tanner stage) was recoded as 1 for stage III and 0 for all other stages. We constructed the final model using stepwise forward regression and expressed its performance in predicting HOMA-IR with adjusted R^2^ values. All calculations were performed with Statistica 13.1 (Statsoft) software.

## Results

### Group characteristics

Among the 5058 subjects aged 6–17 years who were hospitalized in the study period, 174 children/adolescents (45.4% boys) with a median age of 12.10 years were enrolled in the study after taking into account the exclusion criteria and consent to participate. The study included 49 (28.1%) children under 10, the youngest being 6.15 years old. Based on the 85th and 95th BMI percentile cut-offs, the group was divided into participants with normal body weight (*N* = 48, 35.4% boys), obesity (*N* = 92, 50% boys) and overweight (*N* = 34, 47.1% boys). The sex distribution was similar in all three subgroups (*p* = 0.2525).

**Fig. 1:**
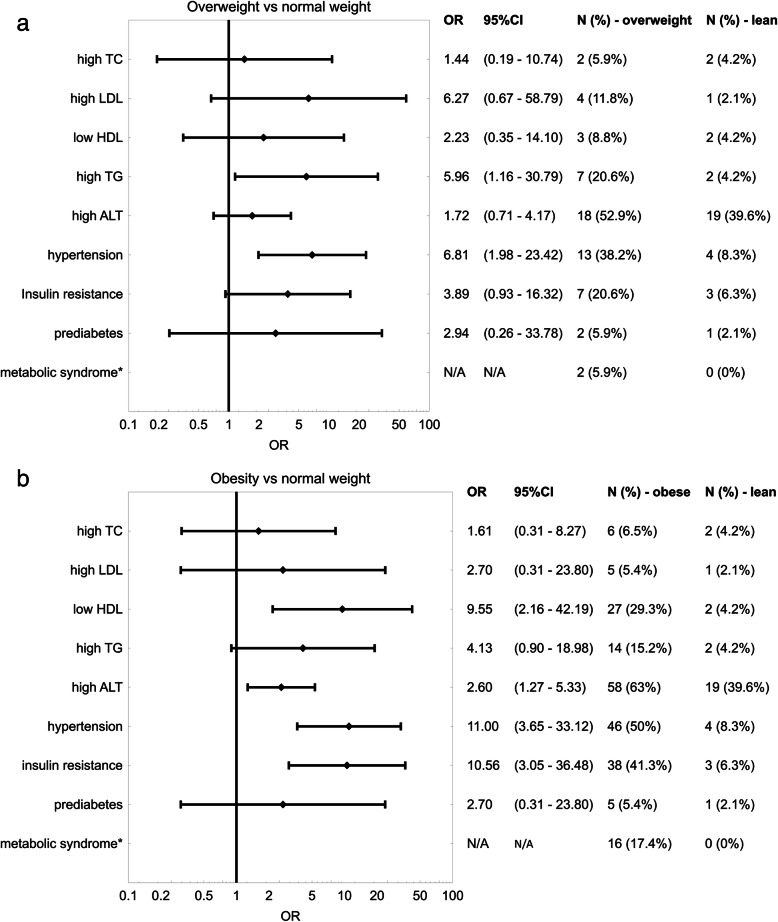
Relationship between overweight (**a**) and obesity (**b**) and odds of developing metabolic abnormalities relative to the same parameters in children with normal body weight. The points indicate odds ratios (ORs) with 95% confidence intervals (95% CIs). TC – total cholesterol. LDL – low-density lipoprotein cholesterol. HDL – high-density lipoprotein cholesterol. TGs – triglycerides. ALT - alanine transaminase

The anthropometric and biochemical features of the studied group are presented in Table 1. Notably, the groups were similar in terms of age (*p* = 0.3812) and pubertal stage (*p* = 0.8710). However, they differed significantly in terms of metabolic conditions. MS was diagnosed in 18 patients (10.3%). The components of MS as well as other abnormalities were more prevalent in overweight and obese patients than in those with normal weight (Fig. 1). The fasting plasma glucose level ≥ 100 mg/dl was confirmed in case of one child (control group). The frequency of impaired glucose tolerance was similar across the groups, and neither being overweight [OR = 2.94 (95% CI: 0.26–33.78)] nor obese [OR = 2.70 (95% CI: 0.31–23.8)] was associated with significantly increased risk. The groups demonstrated significant differences in IR measured by HOMA-IR (Table 1). However, only obesity significantly increased the risk of IR after taking into account the reference value for sex and age [increased in 41.3% of patients with obesity vs 6.3% of those with normal weight, OR = 10.56 (95% CI: 3.05–36.48)] (Fig. 1). The groups also presented significant discrepancies in cardiovascular profiles (Table 1, Fig. 1).

**Table 1:** Characteristics of the study population

Variable	Normal weight (*N* = 48) median (25–75%)	Overweight (*N* = 34) median (25–75%)	Obesity (*N* = 92) median (25–75%)	*p*-value
Age [years]	13.6 (10.1 to 15.7)	12.0 (9.9 to 15.9)	12.0 (9.7 to 14.3)	0.3812
Tanner score	2 (2 to 4)	2 (1 to 4)	2 (2 to 4)	0.8710
BMI [kg/m^2]	18,4 (16.6 to 20.3)	24.1 (22.4 to 25.7)	27.4 (25.9 to 30.6)	N/A
BMI z-score	−0.1 (− 0.6 to 0.4)	1.5 (1.3 to 1.6)	2.1 (1.8 to 2.3)	N/A
BMI percentile	45.8 (26.4 to 66.0)	92.7 (90.5 to 94.3)	98.0 (96.7 to 99.0)	N/A
Body fat [%]	23.9 (20.1 to 27.3)	37.7 (32.9 to 47.0)	45.2 (39.2 to 52.9)	< 0.0001^1^
Fasting glycaemia [mg/dl]	80 (76 to 83.5)	79 (75 to 85)	80 (77 to 84.5)	0.6825
Fasting insulinaemia [μU/ml]	9.1 (5.7 to 13.4)	12 (10.8 to 16.2)	15.9 (9.9 to 25.2)	0.0005^2^
HOMA-IR	1.8 (1.1 to 2.7)	2.5 (2.1 to 3.2)	3.2 (2.1 to 5.1)	< 0.0001^3^
TC [mg/dl]	151.5 (138.0 to 174.0)	161.5 (148.0 to 183.0)	153.0 (135.0 to 175.0)	0.1760
HDL-C [mg/dl]	53.0 (49.0 to 59.5)	47.5 (42.0 to 54.0)	44.0 (38.0 to 51.0)	< 0.0001^4^
LDL-C [mg/dl]	79.0 (69.5 to 97.5)	90.5 (74.0 to 102.0)	89.0 (75.0 to 106.5)	0.1721
TGs [mg/dl]	77.5 (55.5 to 95.5)	103.0 (62.0 to 135.0)	104.5 (79.5 to 128.0)	0.0007^5^
Klotho [pg/ml]	116.6 (38.5 to 163.9)	131.3 (78.0 to 313.0)	168.6 (90.2 to 375.9)	0.0334^6^
FGF19 [pg/ml]	232.8 (126.0 to 340.5)	167.6 (118.2 to 276.6)	160.6 (87.5 to 260.2)	0.0563
FGF21 [pg/ml]	82.4 (31.6 to 128.2)	87.3 (47.1 to 181.4)	89.3 (43.3 to 193.2)	0.3783

*N* number of subjects, *N/A* not applicable, *BMI* body mass index, *HOMA-IR* Homeostatic Model Assessment of Insulin Resistance, *TC* total cholesterol, *HDL-C* high-density lipoprotein cholesterol, *LDL-C* low*-*density lipoprotein cholesterol, *TGs* triglycerides, *FGF19* fibroblast growth factor 19, *FGF21* fibroblast growth factor 21

1 - Post-hoc comparisons significant between normal weight and overweight (*p* < 0.0001), normal weight and obesity (*p* < 0.0001), and overweight and obesity (*p* = 0.0030) groups

2 - Post-hoc comparisons significant between normal weight and overweight (*p* = 0.0261) and normal weight and obesity (*p* < 0.0001)

3 - Post-hoc comparisons significant between normal weight and obesity (*p* < 0.0001) and normal weight and overweight (*p* = 0.0435)

4 - Post-hoc comparison significant only between normal weight and obesity (*p* < 0.0001)

5 - Post-hoc comparison significant only between normal weight and obesity (*p* = 0.0005)

6 - Post-hoc comparison significant only between normal weight and obesity (*p* = 0.0282)

### FGF19, FGF21 and Klotho level analysis

The protein concentrations did not correlate with the age of the children (Klotho, FGF19) (Table 2) and were not associated with sex [Klotho – males 140.6 pg/ml (88.7 to 323.1) vs 136.8 pg/ml (72.0 to 297.0), *p* = 0.9674; FGF19 – males 150.6 pg/ml (85.9 to 299.7) vs 197.7 pg/ml (123.5 to 279.3), *p* = 0.1125; FGF21 – males 85.1 pg/ml (42.2 to 160.9) vs 89.3 pg/ml (42.8 to 174.2), *p* = 0.5915] or pubertal stage (Klotho – *p* = 0.1838; FGF19 – *p* = 0.4569; FGF21 – *p* = 0.1306). The studied proteins were also not associated with one another (data not shown).

**Table 2:** Klotho, FGF19, and FGF21 concentrations in correlation with age, parameters of nutritional status, lipid and glucose profiles, ALT and HOMA-IR

	Klotho	FGF19	FGF21
**Age**	*R* = 0.053, *p* = 0.4848	*R* = 0.064, *p* = 0.3996	*R* = 0.159, *p* = 0.0360
**BMI Z-score**	*R* = 0.188, *p* = 0.0129	*R* = -0.206, *p* = 0.0062	*R* = 0.111, *p* = 0.1423
**BF%**	*R* = 0.210, *p* = 0.0055	*R* = −0.143, *p* = 0.0600	*R* = 0.202, *p* = 0.0020
**ALT**	*R* = 0.184, *p* = 0.0149	*R* = -0.230, *p* = 0.0022	*R* = 0.015, *p* = 0.8413
**TC**	*R* = 0.097, *p* = 0.1997	*R* = −0.040, *p* = 0.5913	*R* = -0.050, *p* = 0.5057
**HDL-C**	*R* = 0.084, *p* = 0.2649	*R* = 0.122, *p* = 0.1076	*R* = -0.212, *p* = 0.0047
**LDL-C**	*R* = 0.211, *p* = 0.0051	*R* = −0.030, *p* = 0.6852	*R* = 0.087, *p* = 0.2504
**TGs**	*R* = -0.014, *p* = 0.8507	*R* = −0.201, *p* = 0.0077	*R* = 0.187, *p* = 0.0131
**Fasting glucose**	*R* = −0,029, *p* = 0,7082	*R* = − 0,096, *p* = 0,2106	*R* = − 0,084, *p* = 0,2726
**HOMA-IR**	*R* = 0.129, *p* = 0.0879	*R* = − 0.313, *p* < 0.0001	*R* = 0.098, *p* = 0.1978

*HOMA-IR* Homeostatic Model Assessment of Insulin Resistance, *BMI* body mass index, *BF%* body fat [%], *TC* total cholesterol, *LDL-C* low-density lipoprotein cholesterol, *HDL-C* high-density lipoprotein cholesterol, *TGs* triglycerides, *ALT* alanine aminotransferase, *FGF19* fibroblast growth factor 19, *FGF21* fibroblast growth factor 21

The protein profiles showed weak associations (Klotho, FGF19) or no association (FGF21) with the body mass of the children. Weak associations were also detected between adiposity and the concentrations of studied markers (Klotho, FGF21) (Table 2). Division by body weight status (normal weight, overweight or obese – Table 1) revealed significant differences in Klotho concentration (*p* = 0.0334). The discrepancy was greatest among those with obesity [median concentration 168.6 pg/ml (90.2 to 375.9)] and normal body weight [median 116.6 pg/ml (38.5 to 163.9)] (post hoc *p* = 0.0282). The differences between overweight and obese patients (post hoc *p* = 1.0000) as well as overweight subjects and subjects with normal body weight (post hoc *p* = 0.3633) were not significant. Furthermore, there were several significant associations of the studied protein concentrations with lipid profiles as well as ALT levels (Table 2).

Among the three proteins, only FGF19 showed a significant association with the HOMA-IR index [FGF19 – *R* = -0.31, *p* < 0.0001] (Table 2). However, dividing children by sex- and physical development-adjusted targets demonstrated that those with IR presented higher concentrations of Klotho [185.3 pg/ml vs 132.6 pg/ml, *p* = 0.0283] and lower concentrations of FGF19 [143.0 pg/ml vs 195.6 pg/ml, *p* = 0.0233] (Table 3).

**Table 3:** Median (IQR) serum values of Klotho, FGF19 and FGF21 in relation to the occurrence of metabolic syndrome and its components as well as insulin resistance and impaired glucose tolerance

	Condition present	Condition absent	*P* value
**Klotho**			
Central obesity (*N* = 116)	**156.4 (89.6 to 350.9)**	**118.5 (48.4 to 177.5)**	**0.0275**
Low HDL (*N* = 32)	156.4 (92.4 to 266.7)	134.4 (75.5 to 335.8)	0.8202
High TG (*N* = 23)	134.2 (90.7 to 321.1)	140.3 (75.5 to 313.0)	0.5620
Hypertension (*N* = 63)	157.5 (90.2 to 321.1)	136.0 (63.9 to 313.0)	0.1975
Metabolic syndrome (*N* = 18)	172.7 (123.0 to 321.1)	135.0 (75.1 to 305.0)	0.2050
Impaired glucose tolerance (*N* = 8)	159.6 (131.8 to 208.3)	136.4 (75.5 to 323.1)	0.4948
Insulin resistance (*N* = 48)	**185.3 (102.1 to 398.2)**	**132.6 (63.9 to 275.6)**	**0.0283**
**FGF19**			
Central obesity (*N* = 116)	**160.6 (92.2 to 260.2)**	**229.4 (119.5 to 345.0)**	**0.0264**
Low HDL (*N* = 32)	147.6 (79.4 to 257.8)	194.2 (112.4 to 289.2)	0.1727
High TG (*N* = 23)	**124.3 (79.3 to 213.8)**	**184.9 (112.4 to 297.8)**	**0.0136**
Hypertension (*N* = 63)	145.7 (93.3 to 244.5)	197.7 (101.1 to 299.7)	0.1085
Metabolic syndrome (*N* = 18)	133.0 (62.2 to 171.2)	186.5 (109.2 to 284.7)	0.0509
Impaired glucose tolerance (*N* = 8)	**86.9 (65.4 to 149.4)**	**183.8 (106.0 to 289.2)**	**0.0416**
Insulin resistance (*N* = 48)	**143.0 (81.5 to 229.3)**	**195.6 (118.2 to 297.8)**	**0.0233**
**FGF21**			
Central obesity (*N* = 116)	**93.0 (48.8 to 199.5)**	**70.1 (31.5 to 108.6)**	**0.0193**
Low HDL (*N* = 32)	100.3 (48.8 to 165.1)	84.7 (42.1 to 161.0)	0.5064
High TG (*N* = 23)	**124.6 (78.6 to 363.5)**	**81.1 (39.5 to 151.3)**	**0.0035**
Hypertension (*N* = 63)	**124.6 (61.3 to 260.3)**	**75.2 (39.4 to 115.3)**	**0.0004**
Metabolic syndrome (*N* = 18)	**136.2 (86.5 to 239.9)**	**82.6 (41.8 to 152.4)**	**0.0286**
Impaired glucose tolerance (*N* = 8)	107.2 (66.2 to 216.8)	86.4 (42.2 to 161.0)	0.4767
Insulin resistance (*N* = 48)	91.9 (44.5 to 159.0)	82.6 (42.2 to 161.0)	0.6314

Numbers in first column represent the number of patients with a given clinical condition. The remainder of the group (174-N) were free from these ailments

MS-metabolic syndrome, FGF19 – fibroblast growth factor 19, FGF21 - fibroblast growth factor 21

Significant differences between patients with or without each condition (in columns) were bolded

Finally, those with MS presented an elevated concentration of FGF21 [136.2 pg/ml vs 82.6 pg/ml, *p* = 0.0286]. FGF19 and FGF21 disturbances were also distinct for particular MS components. FGF21 concentration was markedly elevated in the subjects with arterial hypertension and high TGs levels compared with the concentrations in children with normal blood pressure [124.6 pg/ml vs 75.2 pg/ml, *p* = 0.0004] and normal TGs levels [124.6 pg/ml vs 81.1 pg/ml, *p* = 0.0035]. Central obesity was associated with increased Klotho levels [156.4 pg/ml vs 118.5 pg/ml, *p* = 0.0275] and FGF21 levels [93.0 pg/ml vs 70.1 pg/ml, *p* = 0.0193] as well as a decrease in FGF19 levels [160.6 pg/ml vs 229.4 pg/ml, *p* = 0.0264] (Table 3). The analysis of the multivariate model for HOMA-IR showed that FGF19 was an independent predictor of IR in the studied subjects after adjusting for pubertal stage, sex, age and BMI Z-score (Table 4). Quantitatively, each 100 pg/ml decrease in FGF19 serum concentration was associated with an 8.2% increase in HOMA-IR. This effect was comparable to the impact of physical development (Eta^2^ for FGF19 3.7%, for Tanner stage – 3.8%). The model, however, managed to explain only a small portion of the overall HOMA-IR variation (*R*^*2*^ = 30%).

**Table 4:** Multivariate linear regression for log10(HOMA-IR)

	Multivariate linear regression for log10(HOMA-IR) *R*^*2*^ = 0.30, adj. *R*^*2*^ = 0.28				
	Parameter	95% CI	*p*-value	Eta^2^ [%]	Commentary
**Intercept**	0.278	(0.099 to 0.457)	0.0025	5%	
**Gender - male**	0.006	(−0.037 to 0.038)	0.9731	< 0.1%	Associated with 1.4% higher HOMA-IR compared with girls
**Tanner - stage III**	0.079	(0.018 to 0.139)	0.0112	3.8%	Associated with 19.9% higher HOMA-IR than other stages of puberty
**Age [years]**	0.009	(−0.003 to 0.021)	0.1509	1.2%	Associated with 2.1% higher HOMA-IR for each year
**BMI Z-score** **[standard deviations]**	0.121	(0.086 to 0.156)	< 0.0001	21.3%	Associated with 32.1% higher HOMA-IR for each unit increase in BMI Z-score
**FGF19 concentrations** **[100 pg/ml]**	−0.037	(−0.067 to − 0.008)	0.0124	3.7%	Associated with 8.2% drop in HOMA-IR for each 100 pg/ml increase in FGF19

The constructed model explains a minor fraction (~ 30%) of HOMA-IR variability among the patients, which demonstrates that individual insulin resistance is highly variable and might depend on factors other than those investigated in this study

*HOMA-IR* Homeostatic Model Assessment of Insulin Resistance, *BMI* body mass index, *FGF19* fibroblast growth factor19

R^2^ – proportion of variance in log10 (HOMA-IR) explained by the model

Eta^2^ – proportion of variance in log10 (HOMA-IR) explained by each factor

## Discussion

In our study, we examined the concentrations of circulating FGF19, FGF21 and Klotho proteins among normal weight, obese and overweight children and adolescents and their relationships with metabolic parameters. The results complement the existing reports that thus far lack pediatric-specific data.

We noted increased FGF21 concentrations in children and adolescents with MS compared to the concentrations in other subjects. Moreover, FGF21 levels correlated with both the clinical (adiposity, arterial hypertension) and biochemical (TGs, HDL-C) features of MS. Despite the role of FGF21 in metabolism regulation, reports on its usefulness as a biomarker for obesity and abnormalities associated with MS are conflicting [11, 28–30]. FGF21, produced mainly in the liver during fasting, promotes gluconeogenesis, lipolysis, and ketogenesis; ameliorates glucose uptake; and improves insulin sensitivity [5, 8]. It was previously shown that systemic administration of FGF21 has therapeutic benefits against obesity-related medical complications in obese animals [31–33]. FGF21 analogs tested as antidiabetic drugs in obese and overweight humans reduced dyslipidemia and steatosis. However, no body weight reduction effects were observed [8]. Despite the potential beneficial effects of FGF21, increased endogenous FGF21 levels have been observed in adults with obesity. This paradoxical phenomenon led to the hypothesis that central obesity is a state of FGF21 resistance with compensatory FGF1 overproduction resulting from decreased FGF coreceptor (betaKlotho) expression in white adipose tissue. This hypothesis seems to corroborate our results as well other authors’ previous findings [34, 35]. However, similar to Reinehr et al., we did not confirm the relationship between FGF21 concentrations and insulin resistance [36]. These results may be explained by new data showing that elevated FGF21 levels in individuals with obesity serve as a defense mechanism to protect against systemic IR through upregulation of adiponectin in subcutaneous but not visceral fat, followed by anti-inflammatory action resulting from local M2 macrophage polarization [37].

To our knowledge, this is the first study in children and adolescents to show that FGF19, in addition to pubertal stage and BMI Z-score, is an independent predictor of IR. Given that current reports on the relationship between FGF19 levels and metabolic parameters (including IR) are conflicting [6, 9, 10, 38], our results provide further evidence for discussion.

FGF19 is released from the small intestine in response to food intake and reaches its peak serum level 3 h after a meal compared with 1 h for insulin [5]. In liver cells, FGF19 acts through the FGFR1/betaKlotho or FGFR4/betaKlotho pathway. The activation of the FGFR1/betaKlotho pathway regulates glucose and lipid metabolism. On the other hand, FGFR4/betaKlotho receptor activation is connected with the reduction in bile acid levels and alteration in bile acid pool composition, which may potentially increase TGs levels [5, 6, 39]. When administered to obese mice, FGF19 led to a reduction in body mass, decreased blood glucose levels and increased insulin sensitivity [9, 40]. However, although FGF19 triggers metabolic processes similar to those activated by insulin, the differences between the two hormones are still not well understood [5]. It has been speculated that insulin and FGF19 may have an inverse effect on each other [6]. Consequently, the insulin-resistant state leading to increased levels of circulating insulin may provoke the observed decrease in FGF19 levels. However, it was previously shown that FGF19 production is regulated by numerous other factors, e.g., diet composition, circadian rhythm, antibiotic use, microbiota composition and surgery [9]. Most of these factors were controlled for in our study (e.g., fasting before blood sampling, no antibiotic use, etc.), but FGF19 concentrations observed in the patients were still highly variable. Finally, although we did not check our patients for NAFLD, we measured ALT, which in case of childhood obesity is a recommended screening test for this condition [41]. Interestingly, the results showed a negative correlation between ALT and FGF19 levels in the study groups. These findings are in line with those of Wojcik et al., who suggested that a decrease in fasting FGF19 may be a new important risk factor for NAFLD and MS in adolescents [12].

In our study, we noted that children and adolescents affected by obesity showed higher serum Klotho concentrations than those with normal body weight. This finding is in contrast with other studies on the matter. Amitani et al. showed markedly lower plasma Klotho levels in patients with obesity and anorexia nervosa than in the control group, which suggests that Klotho may reflect normal nutritional status [17]. On the other hand, in a group of healthy Latino neonates, Wojcicki et al. found no association between weight, length at birth or obesity in early childhood and cord blood Klotho levels [19]. Concerning children and adolescents, the literature does not provide sufficient data on the relationship between obesity and Klotho levels in these age groups. Our results may be supported by the fact that Klotho is one of the regulators of adipogenesis. It was previously revealed that Klotho increases adipocyte differentiation in vitro [42]. Moreover, mice without the *Klotho* gene were shown to have less detectable adipose tissue than wild-type animals [16]. Finally, mice that lack the *Klotho* gene are resistant to obesity induced by a high-fat diet [16, 43].

Interestingly, we noted differences in Klotho levels between patients with IR and those with normal insulin sensitivity. A possible explanation is that Klotho participates in the enzymatic modification of N-glycans in insulin and IGF-1 receptors and thus inhibits the intracellular insulin/IGF-1 signaling pathway. As a result, insulin-stimulated glucose uptake becomes blocked, which contributes to IR development [15, 44]. Importantly, inhibition of the IGF-1 signaling cascade is likely associated with increased resistance to oxidative stress and leads to the extension of life, which is one of the major functions of Klotho [45].

There are potential limitations of our study. First, we studied only peripheral hormone levels and did not assess local (i.e., liver, adipose tissue) expression levels, which was out of scope for this study. Second, we relied on BMI Z-score to recognize overweight and obesity without body content assessment by dual-energy X-ray absorptiometry (DXA), as it was not available. Estimated BF% could not be translated into sex- and age-independent Z-scores or percentiles due to the lack of modern pediatric charts for the Polish population. Moreover, serum Klotho concentrations may depend on vitamin D and calcium-phosphate homeostasis, which we did not examine in the studied subjects. However, the abovementioned dependence is observed mostly in patients with chronic kidney diseases, who were excluded from this study. Our multivariate model for HOMA-IR explained only a small fraction of patient-to-patient variability. This demonstrates that there are likely other factors that might be associated with IR in a stronger and more direct way. The subjects were also enrolled in the study in a hospital setting, which might be a potential limitation. However, the inpatient conditions assured a similar exposure to potential confounding factors such as diet, physical activity and ambient temperature. Finally, the samples were taken from the local population, which prohibits us from generalizing the results to Polish or European children.

## Conclusions

In the studied pediatric group, increased serum Klotho concentrations were associated with obesity and IR. This may suggest the existence of a mutual regulation of hormones in the insulin/IGF-1 signaling pathway. We also found a negative association between HOMA-IR and FGF19 concentrations, which may result from the compensatory effect of the interaction between insulin and FGF19. The increased FGF21 concentrations observed in children and adolescents with MS may be an effect of the FGF21 resistance observed in subjects with central obesity.

## Data Availability

The datasets used and/or analyzed during the current study are available from the corresponding author on reasonable request.

## Abbreviations

IR: Insulin resistance
MS: Metabolic syndrome
NAFLD: Nonalcoholic fatty liver disease
FGF19: Fibroblast growth factor 19
FGF21: Fibroblast growth factor 21
BMI: Body mass index
BF%: Body fat %
LDL-C: Low-density lipoprotein cholesterol
TGs: Triglycerides
TC: Total cholesterol
HDL-C: High-density lipoprotein cholesterol
ALT: Alanine aminotransferase
OGTT: Oral glucose tolerance test
HOMA-IR: Homeostatic Model Assessment of Insulin Resistance
